# Comparison Between Drug-Coated Balloon and Stents in Large De Novo Coronary Artery Disease: A Systematic Review and Meta-Analysis of RCT Data

**DOI:** 10.1007/s10557-024-07548-2

**Published:** 2024-01-25

**Authors:** Zhiqiang Ma, Kanling Liu, Yanhui Hu, Xiwen Hu, Binyu Wang, Zhengyi Li

**Affiliations:** Division of Cardiology, Departments of Internal Medicine, Liyuan Hospital, Tongji Medical College, Huazhong University of Science and Technology, Wuhan, 430000 People’s Republic of China

**Keywords:** Drug-coated balloon, Stents, Major cardiovascular adverse events, De novo lesion, Meta-analysis

## Abstract

**Purpose:**

Although a number of studies involving small-vessel de novo coronary disease showed clinical benefits of drug-coated balloons (DCB), the role of DCB in large vessel lesions is still unclear.

**Methods:**

We searched main electronic databases for randomized controlled trials (RCTs) comparing DCB with stents for large vessel de novo coronary artery disease. The primary endpoint was major cardiovascular adverse events (MACE), composite cardiovascular death (CD), myocardial infarction (MI), or target lesion revascularization (TLR).

**Results:**

This study included 7 RCTs with 770 participants. DCB were associated with a marked risk reduction in MACE [Risk Ratio (RR): 0.48; 95% confidence interval [CI]: 0.24 to 0.97; *P* = 0.04], TLR (RR: 0.53; 95% CI: 0.25 to 1.14; *P* = 0.10), and late lumen loss [standard mean difference (SMD): -0.57; 95% CI: -1.09 to -0.05; *P* = 0.03] as compared with stents. There is no significant difference in MI (RR: 0.58; 95% CI: 0.21 to 1.54; *P* = 0.27), CD (RR: 0.33; 95% CI: 0.06 to 1.78; *P* = 0.19), and minimal lumen diameter (SMD: -0.34; 95% CI: -0.72 to 0.05; *P* = 0.08) between groups. In subgroup analyses, the risk reduction of MACE persisted in patients with chronic coronary syndrome (RR: 0.25; 95% CI: 0.07 to 0.89; *P* = 0.03), and patients receiving DCB vs. bare metal stent (RR: 0.19; 95% CI: 0.05 to 0.73; *P* = 0.01). In addition, there was no significant difference between the DCB group and the drug eluting stent group for MACE (RR: 0.69; 95% CI: 0.30 to 1.60; *P* = 0.38).

**Conclusion:**

DCB may be an effective therapeutic option in patients with large vessel de novo coronary artery disease.

**Supplementary Information:**

The online version contains supplementary material available at 10.1007/s10557-024-07548-2.

## Introduction

Coronary artery disease (CAD) is still a major cause of mortality despite major improvements in primary and secondary prevention strategies [1, 2]. Drug-coated balloons (DCB) are a novel treatment strategy for CAD, based on combination therapy of balloon and drug, dilating narrowed coronary lesions and achieving lower rates of restenosis by leaving no metal behind [3]. In fact, the clinical efficacy of DCB has been confirmed in small-vessel disease and in-stent restenosis [4, 5]. However, the role of DCB in large vessel de novo coronary artery disease remains uncertain.

DCB have been investigated for more than ten years in cardiovascular disease [6]. The technique of DCB is direct contact of the antiproliferative drug with the vessel wall via a semicompliant balloon, which could inhibit the proliferation of smooth muscle cells. Compared with stents, DCB have several advantages [7, 8]: 1) DCB may be used in subsets of lesions where stents cannot be delivered or where stents do not perform well, such as in torturous vessels, long diffuse calcified lesions, or bifurcated lesions; 2) The DCB angioplasty requires a short dual antiplatelet therapy duration of only 4 weeks and 3) absence of a stent allows the artery’s original anatomy to remain intact, thereby diminishing abnormal flow patterns.

Recently, several studies comparing DCB with stents in large vessel lesions have been reported but no updated meta-analyses are available [9, 10]. In addition, the higher risk of cardiovascular events in DCB for patients with acute coronary syndrome (ACS) has raised concern in two observational studies [11, 12]. It is still unknown whether this concern extends to randomized controlled trials (RCT). Two previous meta-analyses were performed in large vessels, but data relating to the risk of clinical outcomes are scant [13, 14]. Moreover, these meta-analyses included only a small number of studies and included observational studies, which are prone to ascertainment and selection biases.

The aim of this meta-analysis was to compare efficacy and safety between patients undergoing DCB vs. stents for large de novo coronary lesions in RCT. In addition, to explore a target population that could benefit the most from DCB strategy, subgroup analyses were conducted according to the type of CAD [chronic coronary syndrome (CCS) vs. ACS], and the type of stents [drug eluting stent (DES) or bare metal stent (BMS)].

## Methods

### Search for Relevant Research

A systematic literature search was performed to identify randomized clinical trials (RCTs) that compared DCB with stents in patients with CAD and published between January 2000 and November 2022 in English. Two independent reviewers (Ma and Liu) searched databases, including Cochrane Library, Pubmed, and Embase. Disagreements were discussed with another author (Li) and resolved by consensus. The search strategy included both Medical Subject Headings terms (MeSH-terms) and keywords. The following keywords were used: “drug-coated balloon”, “percutaneous coronary intervention”, “myocardial infarction”, “coronary artery disease” and “randomized”. The meta-analysis was conducted in accordance with the Preferred Reporting Items for Systematic reviews and Meta-Analyses (PRISMA) Statement. The study protocol was submitted to PROSPERO (ID: CRD42022372350).

### Study Selection

There will be no restrictions on stent or DCB type. Pre-specified inclusion criteria were: 1) RCTs of patients receiving DCB versus stents; 2) percutaneous coronary intervention (PCI) for de novo lesions with mean reference lumen diameters ≥ 2.75 mm (This value follows the previous study [20]); 3) The study reported at least one of the following outcomes: myocardial infarction (MI), target lesion revascularization (TLR), cardiovascular death (CD), minimal lumen diameter (MLD) and late lume loss (LLL). Excluded criteria included: 1) Reviews, meta-analyses, case reports, and observational studies; 2) Studies of patients without CAD; 3) Studies that did not report endpoints were excluded; 4) data insufficient. The DCB group included any coronary vessels that underwent only drug-coated balloon angioplasty. Patients in the stent group underwent balloon pre-dilation before stent implantation.

### Outcomes and Definition

The primary endpoint was major cardiovascular adverse events (MACE), as defined by the individual trials (Table S1). Secondary endpoints included individual components of MACE, MLD, and LLL. CD was defined as any death that was not clearly of extracardiac origin, and myocardial infarction. MI was defined according to the fourth universal definition of MI [21]. TLR was defined as any repeat revascularization within the stented or DCB-treated segment. MLD was defined as minimal lumen diameter at the end of follow-up. LLL was defined as the MLD immediately after the procedure minus the MLD at the longest follow-up.

### Data Extraction

Relevant information from eligible studies was extracted using prespecified data collection forms. Two reviewers (Ma and Liu) extracted the following data from each eligible study: author, year, sample size, age, gender, underlying disease, vessel diameter, type of DCB and stents, clinical outcomes (MACE or components of the MACE), angiographic outcomes (MLD or LLL), and duration of follow-up. For the Vos et al*.* study, the 2-year-follow-up data were used to obtain additional information [22]. If data were reported only as median and interquartile range or confidence interval, we followed the Cochrane’s recommendation to approximate the values of mean and standard deviation.

### Risk of Bias Assessment

A risk-of-bias assessment was conducted using the Cochrane Collaboration tool for assessing risk of bias [23]. Risk of bias was assessed independently by some authors (Ma, Liu and Li), and disagreement was resolved by discussion. Each study was evaluated for adequacy of randomization (selection bias), blinding for participants and personnel and statistician responsible for analysis (performance bias), blinding of outcome assessment (detection bias), incomplete outcome data (attrition bias), selective reporting (reporting bias), and other bias (conflict of interest). Each risk of bias was rated as either low, unclear, moderate, or high for the RCT.

### Trial Sequential Analysis

To determine whether the evidence in our meta-analysis is reliable, we performed a trial sequential analysis (TSA) for MACE. When the cumulative z curve crosses the trial sequential monitoring boundary, a sufficient level of evidence for the anticipated intervention effect may have been reached and no further trials need be included. If the z curve crosses none of the boundaries, and the required information size has not been reached, there is insufficient evidence to reach a conclusion. TSA was undertaken with type I error of 5% and type II error of 20% by using a random-effects model for those trials. The information size (n = 917) was calculated using an anticipated intervention effect of RR = 0.48 (the intervention effect obtained from our meta-analysis), and the control event proportion of 7%. TSA was performed using TSA Version 0.9.5.10 Beta.

#### Statistical Analyses

A meta-analysis was performed on the basis of aggregate data in eligible studies reporting comparisons of DCB and stents. Risk ratio (RR) or risk difference with a 95% confidence interval was used as a measure of relative risk for the categorical data, such as TLR, mortality of CD, and mortality of MI. Mean difference (MD) with the 95% CI was calculated as the effect size for endpoint with continuous data, such as MLD or LLL. The Cochrane risk-of-bias tool was used for RCTs. The Cochrane Q test and* I*^2^ index were used to quantify the heterogeneity of each study. We considered heterogeneity to be significant when the *p*-value of the Q-test was < 0.1 or the *I*^2^ statistic was ≥ 50%. Random‐effects model was adopted if there was evidence of heterogeneity. Otherwise, fixed‐effects model was used. Subgroup analyses for the primary and secondary outcomes were performed based on age, sex, diabetes status, hypertension status, and comparators (DCB vs. DES and DCB vs. BMS). The results were displayed as a forest plot. Statistical analyses were conducted following the recommendations of the Cochrane Handbook for Systematic Reviews of Interventions and the PRISMA statement. All data analyses were performed by R software (Version R-4.1.2 for Windows).

## Results

### Study Population

**Fig. 1:**
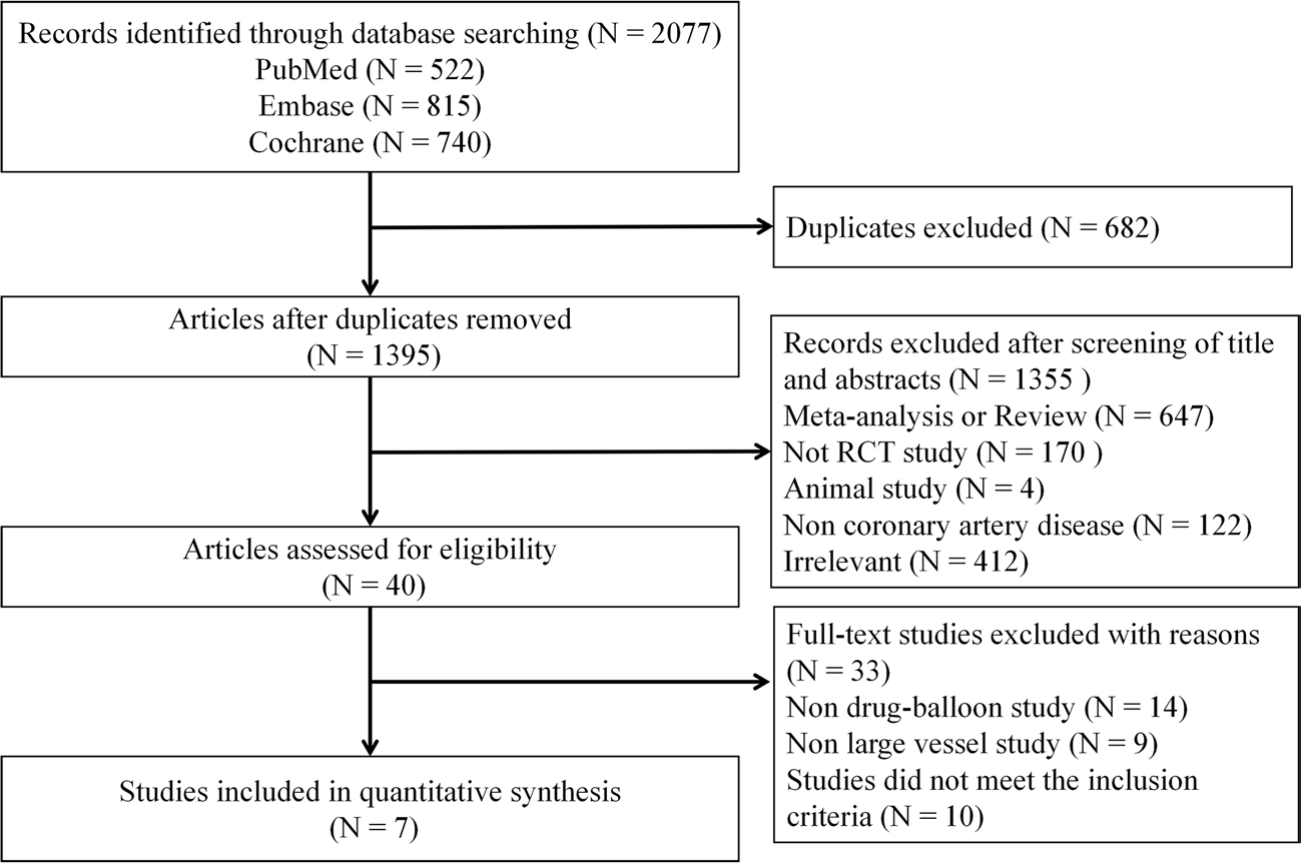
Study search diagram. Summary of how the systematic search was conducted and eligible studies were identified

We identified 2077 studies according to the search strategy. After excluding duplicates, another 1395 studies were excluded according to titles and abstracts. Finally, 7 RCTs were included in our analysis (Fig. 1). Among the 7 included studies, all studies reported clinical outcomes (MACE or components of the MACE). The study of Rissanen et al*.* was powered for MACE. In the study by Rissanen et al*.*, patients were eligible if they had an ischaemic de-novo lesion in a coronary artery and at least one risk factor for bleeding. In the study by Rissanen et al*.,* the average reference vessel diameter was over 2.75 mm in approximately 80% of patients. However, the study by Rissanen et al*.* did not report angiographic outcomes (MLD or LLL).

The cochrane tool was used to assess the quality of all 7 studies. Qualitative evaluation of the included trials revealed overall moderate risk of bias, which was attributable to incomplete description of random sequence generation and assignment concealment, the infeasibility of operator blinding due to manufacturing differences in devices, and the rate of patients lost at clinical and angiography follow-up (Figure S1).

Overall, 770 patients (384 patients randomized to the balloon group and 386 patients randomized to the stent group) were included. The mean age of patients ranged from 49 to 77 years. 512 patients (66%) underwent PCI for ACS. The average reference vessel diameter was over 2.75 mm. More than half of the patients reported hypertension. Follow-up time ranged from 6 to 12 months. In type of stents, two studies (Rissanen et al*.* and Shin et al*.*) including 248 patients used BMS as controls [17, 18]. All types of balloon were paclitaxel-coated or iopromide balloon. In the 7 studies we included, most studies excluded left main coronary artery lesion, severe calcification lesion, or chronic total occlusion. Baseline characteristics of the patients are summarized in Table 1 and Table S2.

**Table 1:** Patient characteristics for all included studies

First author	Year	Patients	N	Age	Treatment	Control	Vessel diameter ^#^	Hypertension ^*^	Diabetes ^*^	Outcomes	FU ^§^
Nishiyama [15]	2016	CCS	60	68	DCB	DES	2.79 ± 0.60	83	41	MLD LLL	8
Gobić [16]	2017	STEMI	75	55	DCB	DES	2.82 ± 0.51	33	8	MACE MLD LLL	6
Rissanen [17]	2019	CCS	208	77	DCB	BMS	> 2.75 (83% patients)	88	37	MACE	9
Shin [18]	2019	CCS	40	59	DCB	BMS	3.45 ± 0.33	42	30	MACE MLD LLL	9
Vos [19]	2019	STEMI	120	57	DCB	DES	3.23 ± 0.49	31	10	MACE MLD LLL	9
Yu [9]	2021	CCS	163	63	DCB	DES	2.88 ± 0.19	63	23	MACE MLD LLL	12
Wang [10]	2022	STEMI	184	49	DCB	DES	3.37 ± 0.52	71	81	MACE MLD LLL	9

^*^ All data are shown as a percent

^#^ All values are measured in millimeters; Values are means ± standard error of the mean

^§^ Units are months

CCS: chronic coronary syndrome, STEMI: ST-segment elevation myocardial infarction, DCB: drug-coated balloon, DES: drug-eluting stent, BMS: bare-metal stent, MLD: minimal lumen diameter, LLL: late lume loss, MACE: major adverse cardiac event, FU: follow-up

### Main Outcomes

#### MACE

**Fig. 2:**
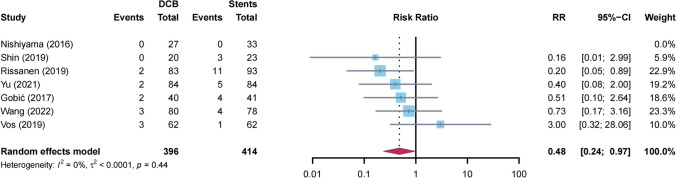
Meta-analysis of major cardiovascular adverse events in patients with large vessel de novo coronary artery disease. The forest plot illustrates the results of the main analysis: DCB compared with stents produced a 52% RR reduction. DCB: drug-coated balloons, CI: confidence interval, RR: risk ratio

MACE were reported in 7 trials (Fig. 2). Patients assigned to DCB showed a 52% risk reduction as compared with those receiving stents (12 vs 28 events; RR: 0.48; 95% CI: 0.24 to 0.97; *P* = 0.04). The statistical heterogeneity was low (*I*^2^ = 6%). Among these studies, the study of Rissanen et al*.* had the highest weight (38.2% of total). Sequential removal of each trial, one at a time, showed that the result became nonsignificant by removing the study by Rissanen et al*.* (*P* = 0.16*)*. Aside from this, other results are stable: the RR ranged from 0.34 (without the study of Vos et al*.; P* < 0.01) to 0.47 (without the study of Shin et al*.; P* = 0.03) (Figure S2). Overall, visual inspection of the contour-enhanced funnel plot for MACE revealed a roughly symmetric distribution (Figure S2).

**Fig. 3:**
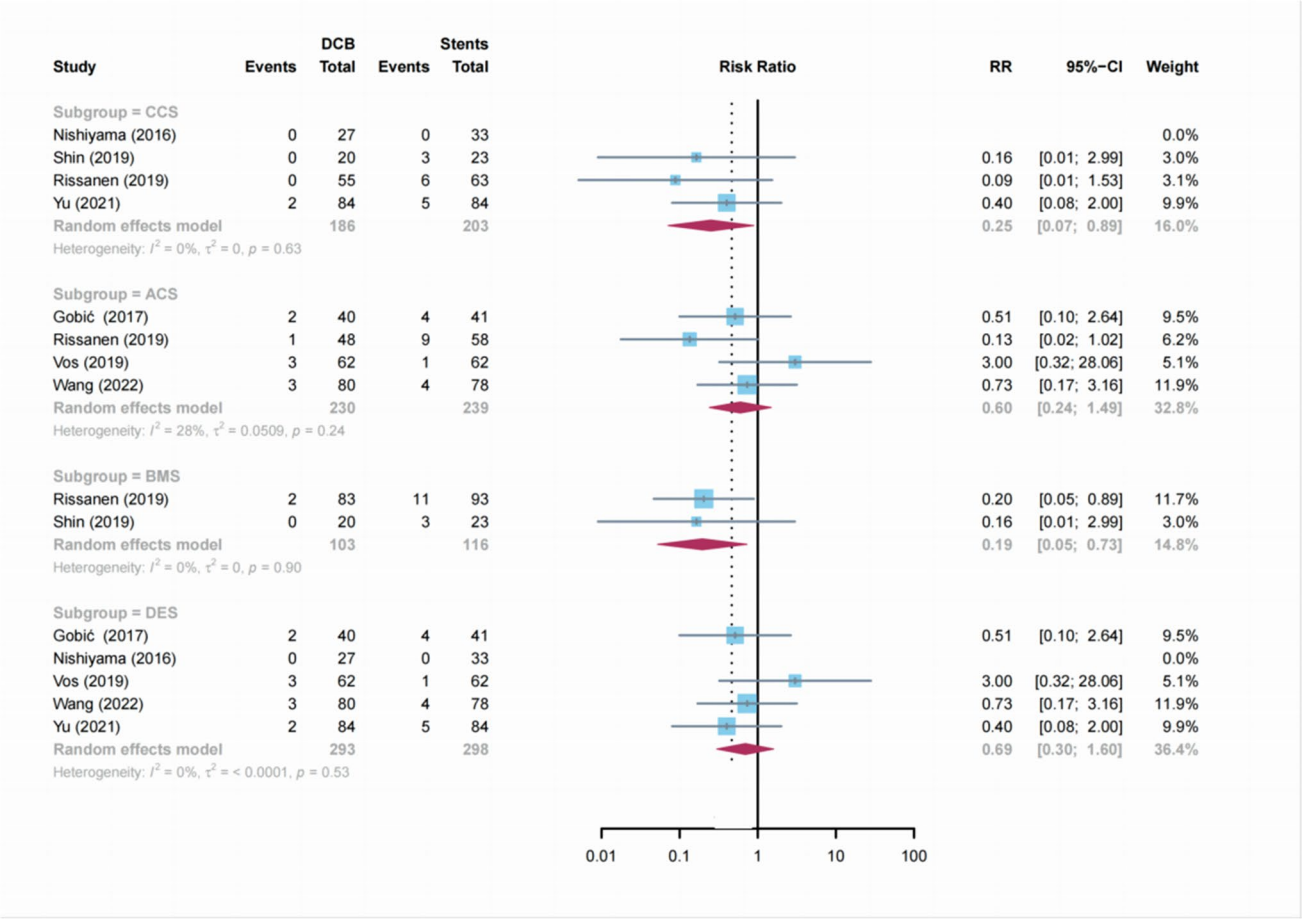
Subgroup analysis of MACE according to patient status and type of stents. DCB: drug-coated balloons, CCS: chronic coronary syndrome; ACS: acute coronary syndrome; BMS: bare-metal stent, DES: drug-eluting stent; CI: confidence interval, RR: risk ratio

Subgroup analysis of the MACE rate was performed to evaluate the effect of type of patient status (ACS vs. CCS). Compared to stents, DCB showed a significant clinical effect only in patients with CCS (2 vs. 14 events; RR: 0.25; 95% CI: 0.07 to 0.89;* P* = 0.03;* I*^2^ = 0%), but not the patients with ACS (9 vs. 18 events; RR: 0.60; 95% CI: 0.24 to 1.49; *P* = 0.26; *I*^2^ = 28%) (Fig. 3). Stratifying the trials according to the type of stents used, we observed a significant clinical effect in the DCB group compared with the BMS group (2 vs. 14 events; RR: 0.19; 95% CI: 0.05 to 0.73; *P* = 0.01; *I*^2^ = 0%), while the trials using the DCB vs. DES were associated with a nonsignificant treatment effect (10 vs. 14 events; RR: 0.69; 95% CI: 0.30 to 1.60; *P* = 0.38; *I*^2^ = 0%) (Fig. 3).

#### TSA

**Fig. 4:**
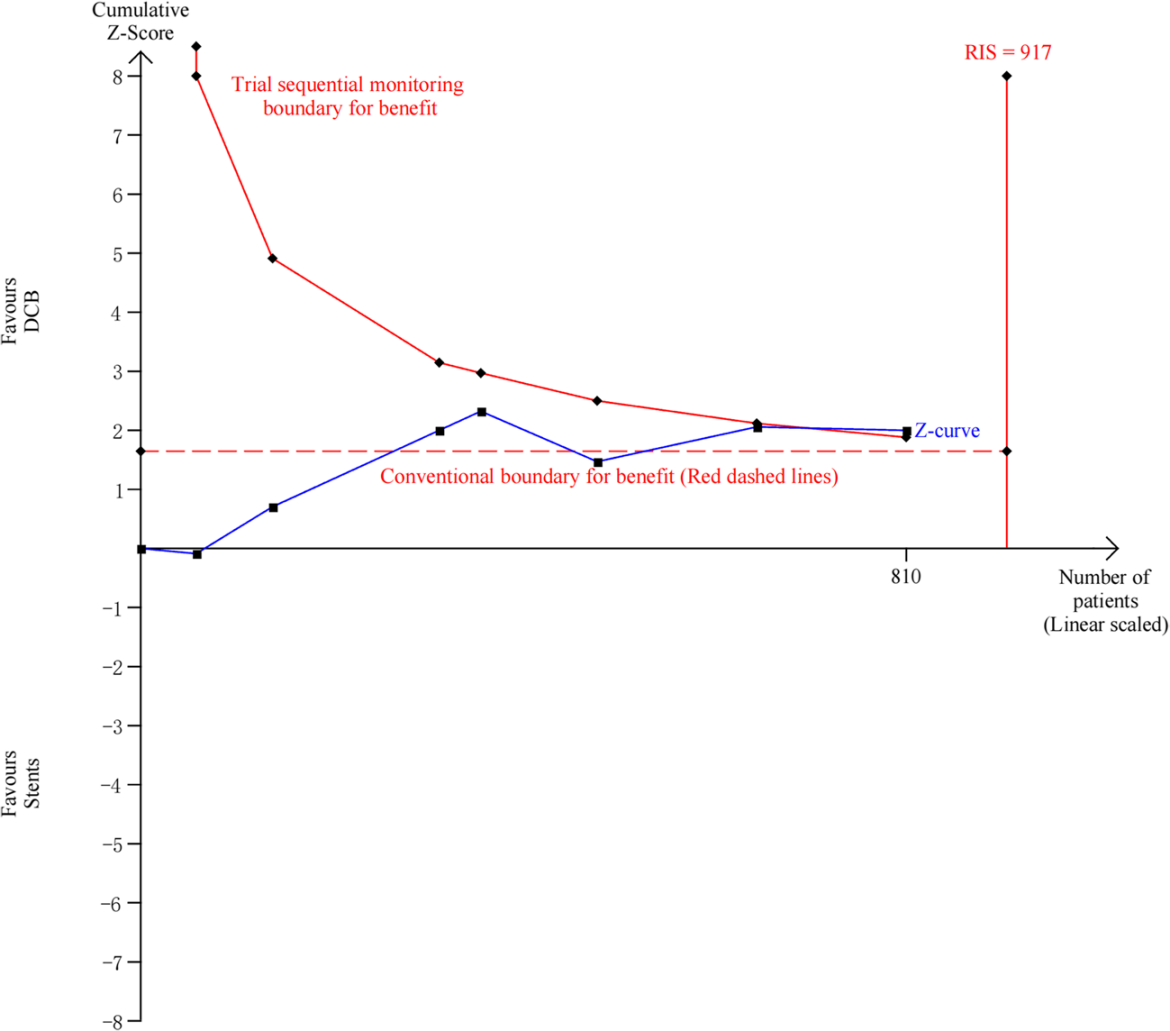
Trial sequential analysis of the included studies. A one-sided graph is plotted by TSA, where the dotted lines represent the conventional significance boundaries, the blue line indicates the cumulative Z-score, and the red lines shows the trial sequential monitoring boundary. TSA, Trial sequential analysis; RIS, Required information size

As shown in Fig. 4, TSA for MACE was conducted using random effects, and the cumulative Z-curve crossed the trial sequential monitoring boundary, but not the RIS boundary. The result of TSA showed the stability of our meta-analysis results.

#### TLR

**Fig. 5:**
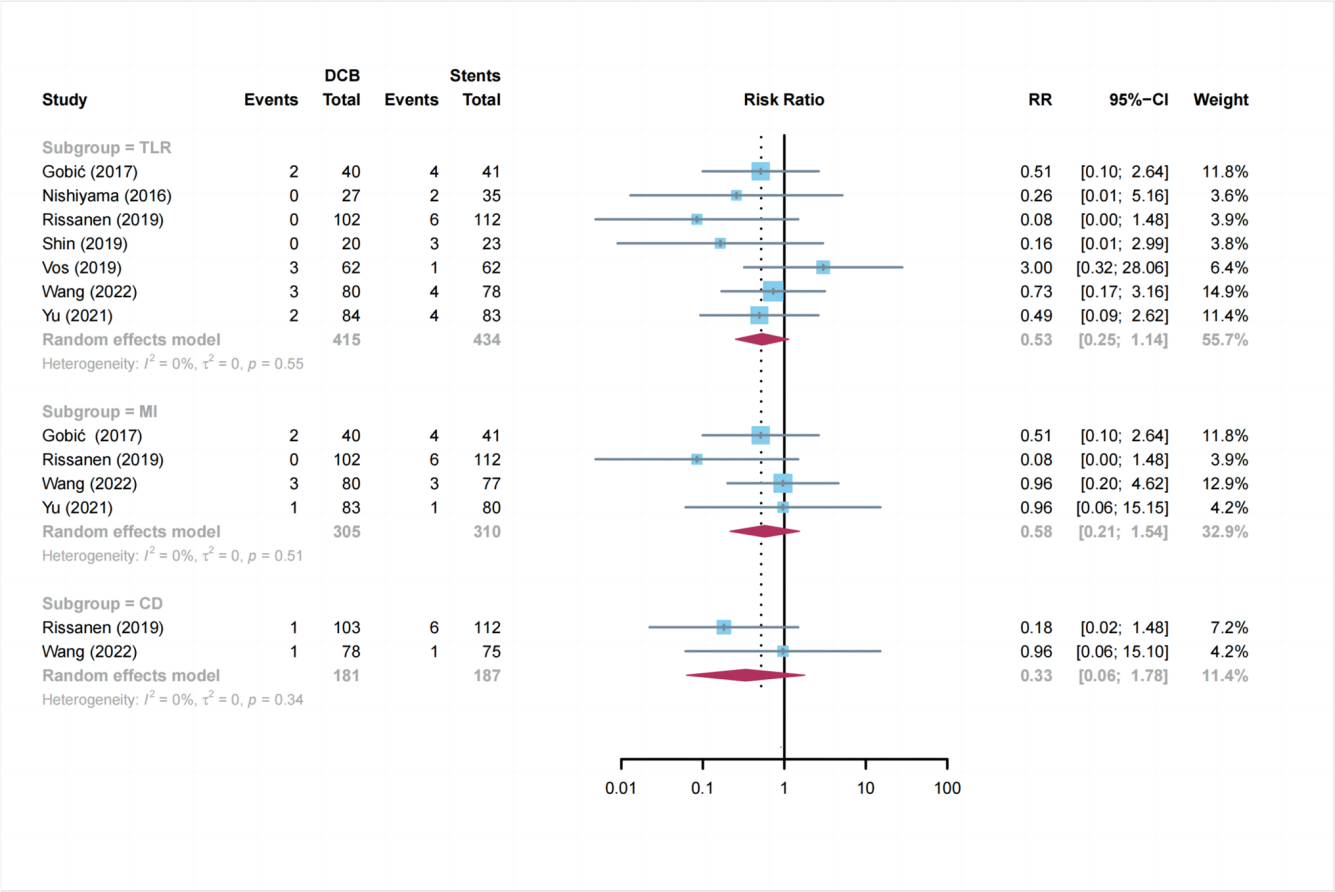
Summary plots for the clinical outcomes. DCB: drug-coated balloon, MI: myocardial infarction, CD: cardiovascular death, TLR: target lesion revascularization, CI: confidence interval, RR: risk ratio

Overall, there were 10 events among 405 patients randomized to DCB and 24 events among 410 patients randomized to stents (Fig. 5). Based on a random-effect meta-analysis, the RR was 0.53 (10 vs. 24 events; 95% CI:0.25 to 1.14; *P* = 0.10; *I*^2^ = 0%).

#### MI and CD

MI event was reported in 4 trials (Fig. 5). Analysis of the pooled data revealed that DCB did not reduce the risk of MI compared with stents (6 vs. 14 events; RR: 0.58; 95% CI: 0.21 to 1.54; *P* = 0.27; *I*^2^ = 0%). In addition, only two studies reported CD events (Fig. 5). Similarly, there was no significant effect observed in CD events (2 vs. 7 events; RR: 0.33; 95% CI: 0.06 to 1.78; *P* = 0.19; *I*^2^ = 0%).

#### LLL

**Fig. 6:**
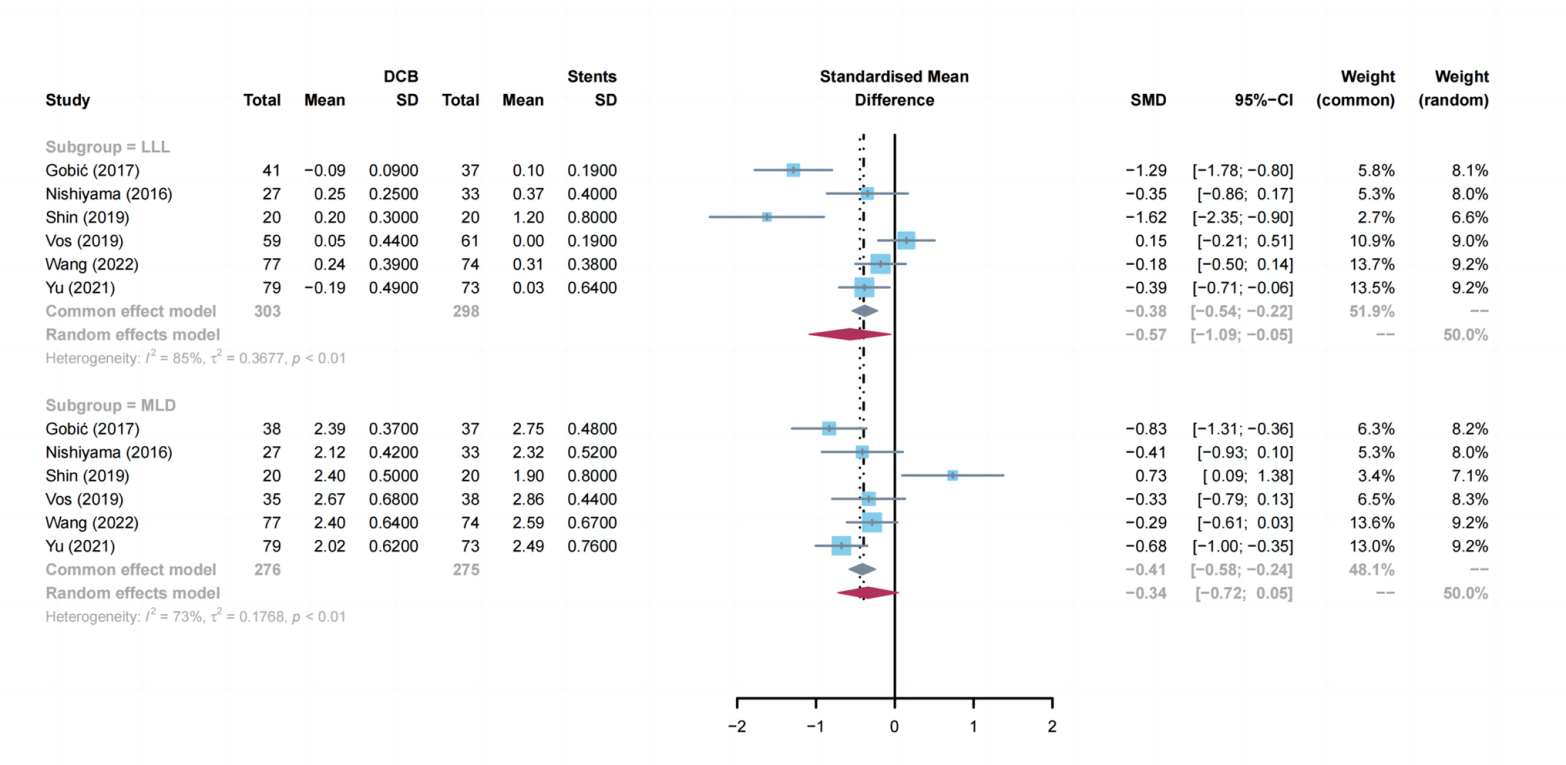
Summary plots for the angiographic outcomes. DCB: drug-coated balloons, MLD: minimal lumen diameter, LLL: late lume loss, CI: confidence interval, RR: risk ratio

LLL event was reported in 6 trials (Fig. 6). Overall, the results showed that LLL in DCB group was smaller than stent group (SMD: -0.57; 95% CI: -1.09 to -0.05; *P* = 0.03; *I*^2^ = 85%). To evaluate the stability of the results, we conducted a sensitivity analysis. We excluded each article in order, but the summary results did not significantly change (*I*^2^ from 79 to 88%). Subsequently, we investigated the possible combinations of trials resulted in an *I*^2^ value below the threshold of low heterogeneity (< 50%). Only by removing the combination of the study by Gobić et al*.*, the study by Vos et al*.* and the study by Shin et al*.*, was heterogeneity low (*I*^2^ = 0%), suggesting that this cluster introduced relevant differences in term of LLL.

#### MLD

Results of the meta-analysis for the MLD are illustrated in Fig. 6. The pooled risk of MLD was similar between the 2 treatments (SMD: -0.34; 95% CI: -0.72 to 0.05; *P* = 0.08; *I*^2^ = 73%;). To analyze the individual impact on heterogeneity, a single trial was removed on at a time, and the individual influence on *I*^*2*^ was estimated. Using this method, we identified that the study of Shin et al*.* was the main origin of heterogeneity. After exclusion of this study, the heterogeneity was significantly decreased, and a significant clinical effect was observed in DCB group compared with stents (SMD: -0.30, 95% CI -0.69 to -0.01; *P* = 0.02; *I*^2^ = 20%).

## Discussion

In this meta-analysis, we observed 5 key findings: 1) DCB would contribute to a decreased risk in MACE compared with stents and this benefit appears to persist in patients with CCS; 2) DCB were associated with a lower risk of MACE and TLR compared with BMS and a similar effect compared with DES; 3) the LLL in the DCB group was lower than the stent group, but there was high heterogeneity between trials; 4) there was a trend but no significant difference in MLD between the DCB and stents; and 5) DCB were associated with no difference in the incidence of CD and MI as compared with stents.

Our meta-analysis differs from prior meta-analyses in several aspects [13, 14]: 1) we found, for the first time, that DCB could reduce the risk of MACE and TLR compared with stents in large vessel de novo coronary lesions; 2) Unlike the previous meta-analysis by Sun et al*.*, a significant difference in MLD between DCB and stents was not observed; and 3) it provides novel insights regarding the available evidence on DCB vs. stents in large vessel de novo lesions.

Although the use of DCB in patients with small vessel disease has been extensively investigated, evidence for the optimal method of revascularization for large coronary lesions remains inadequate. It has been suggested previously that large coronary arteries have more smooth muscle fibers than small vessel arteries and are more prone to recoil and dissection, which may lead to acute occlusion or restenosis of blood vessels [24]. In a retrospective study, although optimal lesion preparation for the use of DCB has been taken, 10% of patients remain need to be bailout stenting due to recoil and dissection [11]. The safety of DCB compared with stents in large vessel disease remain a concern. In this context, by pooling data from 7 studies, our meta-analysis provided some preliminary results for the potential clinical application of DCB in large coronary vessels.

From the subgroup analysis of the MACE according to the type of stents, we observed a significant clinical effect in the DCB group compared with the BMS group (1.9% vs. 13.7%; RR: 0.19; 95% CI: 0.05 to 0.73; *P* = 0.01;* I*^2^ = 0%). Although most patients undergoing PCI are treated with DES, BMS continue to be used in approximately 16% [25]. These patients tend to have high bleeding risk or are uncertain candidates for dual-antiplatelet therapy. Our results provided an important therapeutic strategy for these patients. In addition, DCB did not increase the risk of MACE compared with DES through subgroup analysis (3% vs. 5.5%; RR: 0.69; 95% CI: 0.30 to 1.60; *P* = 0.38; *I*^2^ = 0%). This finding is in accordance with patients with small vessel disease. In BASKET-SMALL 2 trial including 758 patients with de novo in coronary vessels < 3 mm, the rates of MACE are similar in the DCB group and the DES group (15% vs. 15%; *P* = 0.95) [26]. In addition, a similar result was observed in a meta-analysis including 1824 patients with small vessel disease. There was no significant difference between DCB and DES (11.0% vs. 13.5%; *P* = 0.57) [27]. Based on our results, the use of DCB is safe for patients with large vessel disease.

In our study, the incidence of MACE was 3% in DCB and 5.5% in DES, which was much lower than the values in the above studies. There are several possible reasons: 1) In RCTs, 36 patients were not included in the final analysis because the bail-out stenting was forced after the randomization and therefore were excluded from these trials (Table S3). If these 36 patients were included in the final analysis, the incidence of MACE would be about 10%. This result was similar to previous studies [11]; 2) the improvement of operator experience will promote the decrease of bail-out stenting. In clinical practice, secure lesion pre-dilation is absolutely necessary. In the case of unsatisfactory residual stenosis or coronary dissection of Type C or above, bail-out stenting should be performed [3].

The lower incidence of MACE was only observed in patients with CCS (1.2% VS. 8.9%; RR: 0.25; 95% CI: 0.07 to 0.89; *P* = 0.03; *I*^2^ = 0%), but not in patients with ACS (4.0% VS. 8.1%; RR: 0.60; 95% CI: 0.24 to 1.49; *P* = 0.26;* I*^2^ = 28%). Although it has been proposed that DCB in patients with ACS has many potential advantages, such as low risk of thrombosis due to less malposition and homogeneous administration of the drug [28], these advantages did not appear to translate into improved clinical outcomes. In a retrospective registry study, including 487 patients with large vessel coronary lesions, the MACE rate was higher in patients presenting with STEMI (12%) than those with CCS (7%), with p-value less than 0.05 [11]. A rather similar result was also described in a recent study [12]. A mean follow-up of 24 months in the study by Tervo et al*.* showed that the incidence of MACE was significantly higher in patients with STEMI than in patients with CCS (23.9% vs. 7.8%, *P* < 0.01). Because the number of relevant RCT reports is limited, more studies are needed to confirm the efficacy and safety of DCB in patients with CCS or ACS.

Another important finding of this meta-analysis is that the LLL in the DCB group was lower than the stents group (SMD: -0.57; 95% CI: -1.09 to -0.05; *P* = 0.03), with high heterogeneity (*I*^2^ = 85%). The current opinion is that unfavorable remodeling is considered the major determinant of LLL [29]. However, it is unclear whether this remodeling could lead to adverse clinical events. A patient-level meta-analysis including 2426 patients showed that an angiographic LLL ≤ 0.50 mm was not predictive of the incidence of TLR whereas a LLL > 0.50 mm was [30]. This suggested that modest lumen loss does not seem to affect coronary blood flow significantly. Most of the values of LLL in our study are less than 0.5 mm, which suggested that the capability to predict future TLR is very limited. In addition, heterogeneity was not detected by excluding the combination of studies by Gobić et al*.*, Vos et al*.,* and Shin et al*.* (*I*^2^ = 0%). In these studies, patients tend to have fewer (< 50%) risk factors such as hypertension or diabetes. It may be a reason to explain the heterogeneity.

Previous studies had raised concerns about late mortality with DCB [31, 32]. In our analysis, there were no differences in risk of adverse events (MI or CD). This could be for two reasons. 1) the median follow-up considered in this meta-analysis was only 9 months. In such a short amount of time, the exploration of strong but rarer endpoints such as CD or MI is less effective. Longer follow‐up is needed to confirm the efficacy and safety of DCB; 2) patients in this meta-analysis were not only STEMI (57%) but also CCS (43%) on optimal medical therapy with a lower cardiovascular risk, so it is not surprising that in many studies only few death events were registered. As things stand, DCB may be a safe therapeutic option in patients with large vessel de novo coronary artery disease.

Our meta-analysis has several limitations: 1) in this study, different types of DCB were used, such as SeQuent Please DCB, Pantera Lux DCB, and Vasoguard™ DCB. Due to the limited number of included studies, subgroup analysis could not be conducted; 2) there were differences in the core laboratory assessment of the angiographic outcomes (LLL or MLD) between trials. In addition, several studies used different definitions of clinical endpoints in their studies-MACE, TLR, CD, and MI. These may be the reason for the heterogeneity; 3) the lack of patient-level data precluded a careful evaluation for the patient and lesion characteristics that would benefit most from DCBs; 4) This study was limited by the small sample size. Thus, TSA was used to correct these random errors and test for more reliable and conclusive evidence. 5) The majority of the included studies in our analysis excluded left main coronary artery lesion, severe calcification lesion, or chronic total occlusion. Therefore, our results may not be generalizable to these lesions.

## Conclusions

In this meta-analysis of 7 studies comprising 770 patients with large vessel de novo coronary artery disease, DCB were associated with a lower risk of MACE, TLR and LLL compared with stents. In addition, there was no significant difference in the risk of MI and CD between DCB and stents. According to subgroup analysis, the reduction of MACE appears to persist in patients with CCS, and patients receiving DCB vs. BMS. More high-quality research is needed to provide high-quality evidence for further support of DCB efficacy.

## Supplementary Information

Below is the link to the electronic supplementary material.

Supplementary file1 (DOCX 310 KB)

## Data Availability

All data generated or analyzed during this study are included in this article.
