# Supplementary material for: Comparison Between Drug-Coated Balloon and Stents in Large De Novo Coronary Artery Disease: A Systematic Review and Meta-Analysis of RCT Data
Source: Cardiovasc Drugs Ther. 2024 Jan 25;39(3):677–86. doi: 10.1007/s10557-024-07548-2 (PMC12116820; doi:10.1007/s10557-024-07548-2)
Supplement: Supplementary file 1 — Supplementary file1 (DOCX 310 KB) [file 10557_2024_7548_MOESM1_ESM.docx]

Figure S1


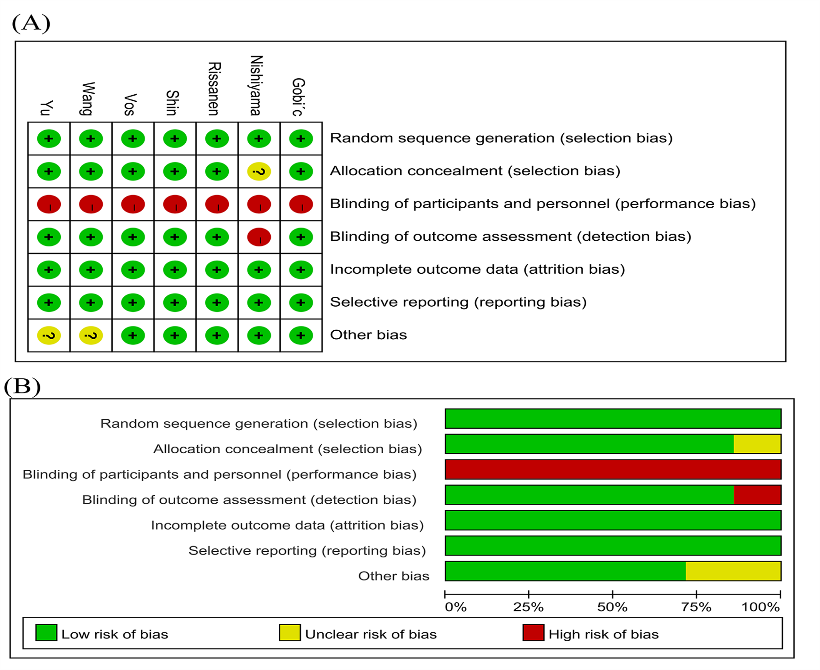


Figure S1: The quality of the included studies.

Figure S2


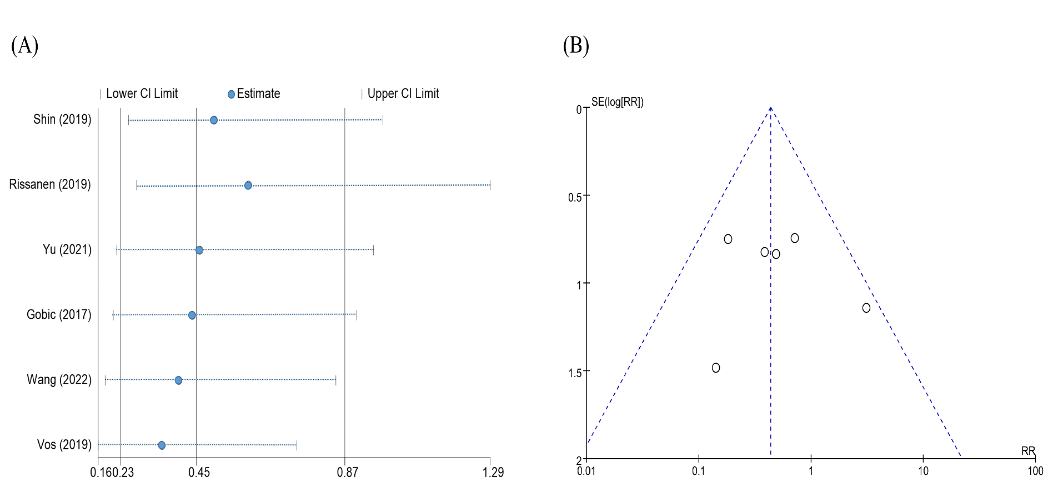


Figure S2: Sensitivity analysis (A) and funnel plot (B) of all included studies.

| **Table S1. Definition of major adverse cardiac events per the individual trials.** | |
| --- | --- |
| First author (Year) | Definition of major adverse cardiac events |
| Nishiyama (2016) | Acute myocardial infarction, congestive heart failure, coronary artery bypass-graft surgery, severe arrhythmia, and stroke |
| Gobi´c (2017) | Cardiac death, non-fatal myocardial infarction, target lesion revascularization, and stent thrombosis |
| Yu (2019) | death, non-fatal myocardial infarction, target lesion revascularization, and target vessel revascularization |
| Rissanen (2019) | Cardiac death, non-fatal myocardial infarction, and target lesion revascularization |
| Vos (2019) | Cardiac death, recurrent myocardial infarction, and target lesion revascularization |
| Shin (2019) | Cardiac death, non-fatal myocardial infarction, target lesion revascularization, and stent thrombosis |
| Wang (2022) | Cardiac death, non-fatal myocardial infarction, and target vessel revascularization and stent thrombosis |

| **Table S2.**  **Major exclusion criteria for studies** | | |
| --- | --- | --- |
| Authors (Year) | | Exclusion criteria |
| Nishiyama (2016) | Left main disease, severe calcification of vessels, chronic total occlusion, and/or extension over 25 mm or more. | |
| Gobić (2017) | Severe coronary artery tortuosity. | |
| Rissanen (2019) | Left main coronary artery lesion, chronic total occlusion, and flow-limiting dissection. | |
| Shin (2019) | Left main disease, multivessel stenosis, chronic total occlusion, and planned coronary artery bypass grafting. | |
| Vos (2019) | Severe calcification vessels. | |
| Yu (2021) | Chronic total occlusion, left main disease, or multiple vessel disease with more than one lesion requiring treatment. | |
| Wang (2022) | Left main coronary artery lesions, >3 coronary diseases, and severely distorted or calcified or angulated vessels. | |
|  | | |

| **Table S3. The summary reasons for exclusion in all studies** | | |
| --- | --- | --- |
| Authors (Year) | Number of patients^$^ | Reasons |
| Nishiyama (2016) | 0 (0%) | There was no lesion for which provisional stenting was needed on account of unsatisfactory DCB balloon dilatation |
| Gobić (2017) | 3 (7%)^*^ | Patients with persistent residual stenosis or type C-F dissection. |
| Rissanen (2019) | 5 (5%)^*^ | Additional lesions were treated with drug-eluting stents in three patients and with bare-metal stents in two patients in the drug-coated balloon-only group. |
| Shin (2019) | 8 (16%)^*^ | Patients had an FFR up to 0.80 or TIMI flow grade up to 2 after balloon angioplasty |
| Vos (2019) | 11 (18%)^*^ | Patients with residual stenosis of >50% (2 cases), coronary artery dissection greater than or equal to type C (8 cases), and crossover to DES treatment (1 case). |
| Yu (2021) | 2 (2%) | Patients with type C dissection |
| Wang (2022) | 9 (4%)^*^ | Patients with type C dissection and/or TIMI < III () |
| * All patients were not included in the final analysis.  $ Percentages are shown in brackets. | | |
